# How does the built environment affect teenagers (aged 13–14) physical activity and fitness? A cross-sectional analysis of the ACTIVE Project

**DOI:** 10.1371/journal.pone.0237784

**Published:** 2020-08-19

**Authors:** Michaela James, Richard Fry, Marianne Mannello, Wendy Anderson, Sinead Brophy

**Affiliations:** 1 Swansea University Medical School, Swansea University, Swansea, United Kingdom; 2 Play Wales, Cardiff, United Kingdom; 3 City and County of Swansea Council, Swansea, United Kingdom; La Inmaculada Teacher Training Centre (University of Granada), SPAIN

## Abstract

Built environments have been cited as important facilitators of activity and research using geographic information systems (GIS) has emerged as a novel approach in exploring environmental determinants. The Active Children Through Individual Vouchers Evaluation Project used GIS to conduct a cross-sectional analysis of how teenager’s (aged 13–14) environments impacted on their amount of activity and influences fitness. The ACTIVE Project recruited 270 participants aged 13–14 (year 9) from 7 secondary schools in south Wales, UK. Demographic data and objective measures of accelerometery and fitness were collected from each participant between September and December 2016. Objective data was mapped in a GIS alongside datasets relating to activity provision, active travel routes, public transport stops, main roads and natural resources. This study shows that fitness and physical activity are not correlated. Teenagers who had higher levels of activity also had higher levels of sedentary time/inactivity. Teenagers showed higher amounts of moderate-to-vigorous physical activity if their homes were closer to public transport. However, they were also more active if their schools were further away from public transport and natural resources. Teenagers were fitter if schools were closer to natural resources. Sedentary behaviour, fitness and activity do not cluster in the same teenagers. Policymakers/planning committees need to consider this when designing teenage friendly environments. Access to public transport, active travel, green space and activities that teenagers want, and need could make a significant difference to teenage health.

## Introduction

Despite the well-documented physiological and psychological benefits of physical activity, many young people are not sufficiently active. Global guidelines recommend 60 minutes of moderate to vigorous physical activity (MVPA) per day in the form of play, games, sports, active travel or planned exercise [1]. However, it is reported that 80% of young people (5–17 years old) are not meeting this, with girls less active than boys [2]. In Wales, only 11% of girls and 20% of boys are sufficiently active [3]. Research suggests this may be because girls have different motivations and barriers to being active [4, 5].

The United Nation’s Convention on the Rights of a Child (UNCRC) [6] defines a child as being anyone under the age of 18. Therefore, the rights of children also apply to teens [7]. In particular, Article 31 of the UNCRC states that every child has the right to play. ‘Playing’ receives a lot of attention in the early years, however provision needs to be made for older children [7].

Accessibility (e.g. lack of active travel routes) and lack of local activity provision have been reported as the main barrier to being active for young people [8–10]. Particular attention has been paid in the literature to transport infrastructure and the location and quality of community resources (e.g. parks/greenspace and activity providers) using freely available map data from commercial points of interest such as food outlets and physical activity provision [11].

Research using objective measures of physical activity via accelerometry has shown the number of park spaces, multi-use pathways (e.g. pavements for walking and cycling) and gyms in local neighbourhoods influences physical activity levels [4, 12, 13]. Analysis of distances from homes to activity enabling spaces has suggested that being within walking distance of these amenities is beneficial for teenage health and fitness [14]. Research shows that girls need to live closer to these provisions to experience benefits [4]. This may be because girls have less independent mobility than boys [4]. However, independent mobility is decreasing in teenagers as a population [15] and therefore, supportive environments and local activity should be valued and considered when planning interventions to promote activity in young people [16, 17].

Research into activity enabling environments has been made easier due to advances in methodological tools such as Geographic Information Systems (GIS). As a result, the number of studies exploring environmental determinants of physical activity has grown [9, 11]. However, research using GIS to predict physical activity and fitness is still in its infancy and to date little research has examined the association between objectively measured environmental variables and teenage physical activity [12]. There is even less research on the impact on fitness and motivation to be active.

The Active Children Through Individual Vouchers Evaluation (ACTIVE) Project [18] aims to add to this literature by using GIS to conduct a cross-sectional analysis of how teenager’s (aged 13–14) environments impact; i) physical activity levels (using accelerometers) and ii) fitness levels of teenagers (using the cooper run test [CRT]). This paper explores a young person’s home and school deprivation levels, objective measures of distances to activities, active travel infrastructure, public transport and distance to natural resources as potential facilitators of activity, as well as fitness levels and motivation to be active. Data on these dependent variables is from the baseline data collection of the ACTIVE Project. The aim of this paper is to provide insight into how policy-makers and activity providers can better facilitate teenage physical activity.

## Materials and methods

The ACTIVE Project recruited 270 participants from year 9 (aged 13–14) from seven secondary schools in south Wales, UK [18]. The methodology for ACTIVE has been previously published and further explanation about the measures can be found in the protocol paper [18]. The College of Human and Health Science Ethics Committee at the College of Medicine, Swansea University granted the ACTIVE Project ethical approval. Demographic data and objective measures of accelerometry, fitness and self-reported motivation were collected from each participant between September and December 2016. This time of year was selected as it was the beginning of the school year and gave a baseline measure prior to significant engagement with school sport and the physical education curriculum which could have affected activity and cardiovascular fitness.

### Data collection

Accelerometery including MVPA and inactivity/sedentary behaviour was measured via Actigraph GT3X+ accelerometers [18]. To improve wear compliance, the accelerometers were worn on the non-dominant wrist of the participant [19, 20]. Apart from bathing or swimming, participants were asked to wear their accelerometer for 7 full days and set to record at a frequency of 30Hz [18].

Cut points for activity were taken from Chandler et al. [21]; sedentary behaviour was defined as periods with counts (the unit of measurement for activity used by Actigraph) below 305 counts per 5 seconds, light activity as 306–817 counts and MVPA defined as periods with counts >818. To be included in the analysis, participants needed to wear the accelerometers for at least 500 minutes on 3 or more days. Periods of >60 minutes with 0 count values was classified as non-wear time and excluded from analysis. This criteria is in line with previous studies [22, 23]. Sedentary behaviour was also included in the analysis.

This study used the Cooper Run Test (CRT) to assess fitness [24]. The CRT is a 12-minute walk/run test where participants were asked to complete as many laps of a school sports hall as possible in the time [18]. The area to run was marked out by the researchers to ensure participants would complete the full lap. The facilities used to run the CRT differed in size depending on the school’s provision and access to space. The number of laps was then converted into a total distance score (in metres). The CRT was carried out during physical education (PE) lessons during school time to avoid disruption [18]. Therefore, participants were split in to two groups so that one group could complete the CRT while the other group recorded scores.

Participants were asked to complete the modified Behavioural Regulation in Exercise Questionnaire (BREQ-2) to measure their motivation to exercise [25] prior to completing the CRT. The 19-item questionnaire provided a total motivation score which was used to define pupils as ‘autonomous’ or ‘controlled’ via five subscales; amotivation, external regulation, introjected regulation, identified regulation and intrinsic motivation [26]. The mean of the five subscales forms an idea of whether teenagers are motivated more autonomously or controlled [26]. In this study, the Relative Autonomy Index (RAI) was used to gain insight into the degree of autonomy the teenagers had. This was calculated by weighing each of the subscales and summing the weighted scores; the minimum score of RAI is -24 and the maximum is +20 [26]. In line with Self-Determination Theory (SDT) [27] being more autonomously motivated means that teenage participation in activity is attributed to enjoyment and personal values as opposed to controlled motivation and being made to feel guilty and external pressure to be active [28].

Deprivation scores for both the home and school was from the Welsh Index of Multiple Deprivation (WIMD) [29]. WIMD is based off numerous indicators such as income, access to services, safety, housing and education [29]. The continuous scale was used in analysis with 1 equating to the most deprived area and 1909 to the least deprived [29].

Objective data was mapped in a GIS alongside datasets relating to activity provision, active travel routes, public transport stops, main roads and natural resource. Lle [30], a geo-portal for Wales which is a partnership between Welsh Government and Natural Resources Wales, was used to access open source maps and create these datasets. Participant homes and schools and nearest Euclidean distance were measured from each school and home location to services and provision using QGIS 2.18. This created a database which was exported for statistical analysis.

### Statistical analysis

Multivariate linear regression models were estimated in STATA (Version 15). Three models were created to answer how the environment influences a) MVPA and, b) fitness. Prior to conducting the analysis, the data was cleaned to remove any readings that were not required for analysis (i.e. METS/kcals) and any outliers, in particular accelerometry measures which did not meet wear time. Assumptions underlying regression models were confirmed and model fit. Checks of random variation of residuals was undertaken for all models. S1 Table presents the correlation data between the independent variables.

Regression models used to assess the associations of variables in the GIS dataset on MVPA, as well as fitness and motivation (level of significance = *p* < 0.05). Sex was included in the models as physical activity and fitness levels in particular differ by sex. Relationships between the environment and physical activity was shown via structural equation modelling (SEM) in STATA and also fitness and motivation as secondary aims.

## Results

Demographic data (Table 1) shows that there were more boys than girls in the study population (62%). In accordance with previous findings, boys were significantly fitter than girls. Yet, they were not statistically more active (using accelerometer data). Distances to active travel, public transport, main roads, natural recourses and activity providers were similar for the participant’s homes and schools on average showing that these built environments have similar provisions. However, boys lived further away from school or from a main road compared to girls.

**Table 1:** Demographics of participants.

	Total		
**Sex**	n = 247		
Boy	n = 152 (62%)		
Girl	n = 95 (38%)		
**Percentage Meeting 60 Mins MVPA**	69% (n = 169)		
Boy	64% (n = 109)		
Girl	36% (n = 60)		
	**Mean (SD)**	**Min**	**Max**
**MVPA (Minutes)**	69.3 (18.4)	26.1	140.5
Boy	70.1 (18.7)	26.1	140.5
Girl	67.9 (18.1)	33.1	126.7
Difference	-2.2 (95% CI: -7.0 to 2.4)		
**Light Activity (Minutes)**	207.4 (46.9)	111.8	552.7
Boy	201.6 (41.4)	111.9	343.5
Girl	205.9 (39.0)	125.9	281.4
Difference	4.3 (95% CI: -10.2 to 18.8)		
**Sedentary Time (Minutes)**	595.7 (89.3)	256.5	798.5
Boy	609.1 (91.2)	256.5	798.5
Girl	574.2 (82.2)	283.1	773.3
Difference	-34.9 (95% CI: -57.4 to -12.2)*		
**Fitness (Metres Ran)**	1840.3 (393.8)	476	2883
Boy	1967.5 (407.2)	476	2883
Girl	1636.7 (267.1)	984	2430
Difference	-330.8 (95% CI: -423.5 to -238.1)*		
**Motivation (Total)**	10.0 (4.7)	-7.9	18
Boy	9.9 (4.4)	-6.7	18
Girl	10.1 (5.2)	-7.9	18
Difference	.2 (95% CI: -1.0 to 1.3)		
**Home Deprivation (WIMD)**	664.9 (559.6)	3	1878
Boy	680.7 (573.9)	3	1878
Girl	639.5 (538.0)	3	1799
Difference	-41.2 (95% CI: -185.6 to 103.1)		
**Home Distance to Active Travel (Metres)**	1438.2 (999.9)	85.1	5217.6
Boy	1425.8 (967.7)	141.5	4933.6
Girl	1458.1 (1054.3)	85.1	5217.6
Difference	32.2 (95% CI: -225.7 to 290.4)		
**Home Distance to Public Transport (Metres)**	143.2 (562.8)	16.4	8879.9
Boy	110.9 (79.3)	16.4	487.9
Girl	195.0 (902.5)	17.9	8879.9
Difference	84.1 (95% CI: -60.8 to 229.0)		
**Home Distance to Main Road (Metres)**	644.5 (454.6)	11.2	3617.3
Boy	662.2 (489.6)	35.5	3617.3
Girl	616.1 (393.0)	11.2	1969.4
Difference	-46.1 (95% CI: -163.3 to 71.0)		
**Home Distance to Natural Resource (Metres)**	1336.6 (752.1)	48.1	4271.4
Boy	1392.2 (797.2)	110.5	4271.4
Girl	1247.7 (668.2)	48.1	3123.3
Difference	-144.5 (95% CI: -337.7 to 48.8)		
**Home Distance to Activity Provider (Metres)**	1108.2 (1324.5)	0	14702.7
Boy	1137.0 (1159.6)	0	13301.7
Girl	1062.1 (1558.1)	0	14702.1
Difference	-74.9 (95% CI: -416.6 to 266.9)		
**Home Distance to School (Metres)**	2321.8 (2349.5)	95.1	20899.2
Boy	2563.0 (2623.4)	95.1	20899.2
Girl	1936.1 (1773.9)	228.5	13587.2
Difference	-626.9 (95% CI: -1228.2 to -25.5)*		
**School Deprivation (WIMD)**	673.3 (674.6)	56	1660
Boy	681.4 (679.7)	56	1660
Girl	660.2 (669.6)	56	1660
Difference	-21.2 (95% CI: -195.2 to 152.9)		
**School Distance to Active Travel (Metres)**	1361.9 (662.1)	596.9	2729.6
Boy	1420.4 (641.4)	596.9	2729.6
Girl	1268.3 (686.9)	596.9	2729.6
Difference	-152.1 (95% CI: -321.9 to 17.6)		
**School Distance to Public Transport (Metres)**	105.2 (66.1)	42.7	276.4
Boy	102.8 (57.7)	42.7	276.4
Girl	108.9 (77.8)	42.7	276.4
Difference	6.1 (95% CI: -10.9 to 23.1)		
**School Distance to Main Road**	800.8 (382.3)	273.5	1654.1
Boy	885.0 (396.5)	273.5	1654.1
Girl	666.1 (316.5)	273.5	1654.1
Difference	-218.8 (95% CI: -313.6 to -124.1)*		
**School Distance to Natural Resource**	1712.9 (569.4)	398.8	2315.1
Boy	1723.4 (591.9)	398.8	2315.1
Girl	1696.1 (533.9)	398.8	2315.1
Difference	27.3 (95% CI: -174.2 to 119.5)		
**School Distance to Activity Provider**	901.4 (891.4)	0	2494.2
Boy	889.9 (905.2)	0	2494.2
Girl	919.7 (873.3)	0	2494.2
Difference	29.8 (95% CI: -200.3 to 259.8)		
*While the averages are different; the minimum and maximums are the same for school distances for boys and girls as it reports on 7 schools*. *because it is a fixed site*, *not individual like the participant’s homes*.			

WIMD = Welsh Index of Multiple Deprivation

*Indicates significance.

Due to not meeting the inclusion criteria of accelerometry wear time (>500 minutes per day), 23 participants were excluded from the analysis (n = 247).

### Moderate-to-vigorous physical activity (MVPA)

Table 2 (R^2 = 0.19) shows that teenagers showed higher MVPA levels if their homes were closer to public transport. Conversely, they were also more active if their schools were further away from public transport and natural resources. Interestingly, teenagers who had higher levels of activity also had higher levels of physical inactivity, which shows a contrasting relationship between MVPA and inactivity. In this study, over 60% of teenagers met government’s recommendations of 60 minutes of MVPA per day on average across the week.

**Table 2:** Linear regression results for MVPA.

Variable	Coef.	95% Confidence Interval	p-value
Sex (Boy = 1)	1.157	-5.570 to 7.884	0.735
Home Deprivation	-.0020	-.006 to .002	0.384
Home Distance to Active Travel Route	-.0008	-.003 to .001	0.525
Home Distance to Public Transport	-.004	-.009 to -.003	0.036*
Home Distance to Main Road	.002	-.003 to .007	0.449
Home Distance to Natural Resource	-.002	-.006 to .001	0.165
Home Distance to Activity Provider	-.0003	-.002 to .001	0.685
Home Distance to School	.0001	-.000 to .001	0.785
School Deprivation	.022	-.005 to .050	0.119
School Distance to Active Travel Route	-.014	-.036 to .007	0.186
School Distance to Public Transport	.189	.047 to .331	0.009*
School Distance to Main Road	.004	-.010 to .019	0.555
School Distance to Natural Resource	.014	.0003 to .029	0.044*
School Distance to Activity Provider	-.010	-.024 to .002	0.120
Fitness (Distance Ran in Cooper Run)	.0004	-.008 to .009	0.927
Sedentary Time	.050	.024 to .076	0.000*
Motivation	-.133	-.619 to .353	0.590

*Indicates significance.

S2 and S3 Tables show that in terms of MVPA there are some small differences between sex. Girls were more active if they were from more deprived homes and their homes were closer to public transport (S3 Table). Boys were more active if their schools were further away from public transport and natural resources. Boys were also more active if they had higher time spent inactive (S2 Table).

### Cardiovascular fitness

Table 3 (R^2 = 0.27) shows boys had higher levels of fitnes*s*. Teenagers were fitter if schools were closer to natural resources, which is in contrast to findings regarding activity levels. Teenagers were fitter if they had higher motivation.

**Table 3:** Linear regression results for cardiovascular fitness (metres ran).

Variable	Coef.	95% Confidence Interval	p-value
Sex (Boy = 1)	474.997	403.550 to 546.444	0.000*
Home Deprivation	.046	-.017 to .111	0.157
Home Distance to Active Travel Route	-.002	-.037 to .032	0.868
Home Distance to Public Transport	.003	-.058 to .066	0.902
Home Distance to Main Road	-.022	-.098 to .053	0.565
Home Distance to Natural Resource	-.008	-.060 to .043	0.761
Home Distance to Activity Provider	-.0007	-.024 to .022	0.950
Home Distance to School	-.008	-.024 to .008	0.326
School Deprivation	-.272	-.670 to .124	0.177
School Distance to Active Travel Route	.110	-.194 to .416	0.475
School Distance to Public Transport	-1.481	-3.494 to .532	0.149
School Distance to Main Road	.160	-.044 to .366	0.124
School Distance to Natural Resource	-.217	-.419 to -.016	0.034*
School Distance to Activity Provider	.149	-.038 to .337	0.119
MVPA	.085	-1.748 to 1.919	0.927
Sedentary Time	-.361	-.764 to .010	0.057
Motivation	7.196	.414 to 13.977	0.038*

*Indicates significance.

S4 and S5 Tables show very little influenced boys’ fitness but for girls, attending a school further away from a main road and having higher motivation had a significant relationship with being fitter (S5 Table).

### Path analysis

**Fig 1:**
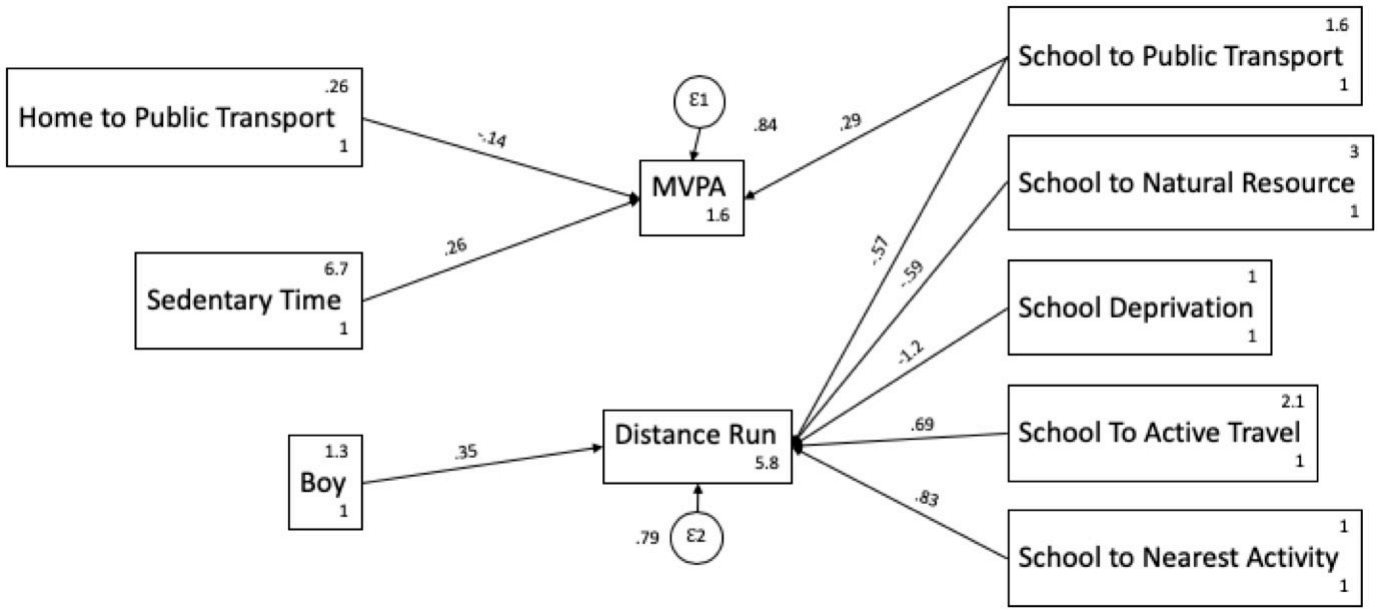
Path analysis model of predictors of MVPA and fitness in teenagers.

Combining all variables using a path analysis model (Fig 1) showed the there was no relationship between levels of MVPA and fitness. The school environment appears integral to fitness; an increasing distance to natural resource and public transport shows a negative effect on fitness. Whereas being further away from active travel and the nearest activity provision shows higher fitness levels. Being more active was influenced by distance to public transport. Teenagers who showed higher levels of MVPA also showed higher inactivity, but inactivity did not affect fitness.

## Discussion

The impacts of the built environment on a teenager’s physical activity/fitness are not straightforward according to this study. Fitness was associated with going to school near natural resources (e.g. green space). While associations between natural resources and PA have been positive [4, 12, 13], this finding shows there may also be associations with fitness too. This is an important link to make as it highlights the cardiovascular benefits to being nearer natural resource for young people and should be considered when planning environments.

In terms of natural resources around schools, the finding that increased proximity improves fitness could mean that some schools’ PE/break time provision is better suited to improving fitness due to better space/resources as highlighted in previous research [31]. For example, these school may have increased access to green space for opportunities to participate in wide range of sports across the PE curriculum. They could also facilitate more outdoor, active classrooms. Thus, improving fitness in a variety of ways.

Moreover, these are spaces that teenagers could use their bikes/scooters safely away from roads or play with their friends in green spaces after-school. These spaces could facilitate forms of more structured sport (e.g. football/rugby) or encompass less conventional activities such as den building [7]. Schools with better access to natural resource may be in less urban/built-up areas thus, further away from high traffic, main roads. Research has attributed roads to impacting activity [9] as safety concerns over traffic are cited in previous studies as reasons why young people are inactive in their communities [32]. These characteristics may encourage inherently more physically active families to live nearby or send their children to schools near natural resource as they are perceived to be conducive to an active lifestyle [32]. This could also underpin the finding that natural resource proximity influences fitness.

When building schools or planning existing school development, this study shows that the school grounds are integral to providing opportunities for better fitness and activity. Currently, school grounds are under-utilised for child-led play and activity when the teaching day ends [33]. Whilst the importance of community access to schools, particularly in more deprived communities is recognised [34], the focus for teenagers is often on adult led and structured activities. Previous studies show this is not what teenagers want and need from provision [5].

There are other findings to consider. Firstly, despite declines in fitness, activity levels actually improve when public transport and natural resource is further away from schools, especially for boys. It could be that the increased walking distance to access these provisions increases activity; suggesting walking can contribute to moderate-to-vigorous activity. This finding highlights that supportive environments and local activity, so teenager’s would not need to travel, should be valued to improve their fitness [16, 17] but activity levels can be sustained despite distance. Conversely, this study also suggests that activity time improves if teenagers lived closer to public transport and went to schools closer to active travel routes. Therefore, giving them the opportunity to travel independently to activity provision/school without the help of parents/guardians.

Previous analysis has shown that being within walking distance of provision is beneficial for teenage fitness [4, 14] and therefore, the importance of considering the needs of teenagers when planning environment’s should not be overlooked. It would seem that teenagers value enjoyment from activity and therefore willing to travel further to do things they like [28] rather than the convenience of accessing whatever activity is on your doorstep or it is simply a case that there is nothing for them to do in their local communities. Access to provision teenagers like may be more relevant for girls, who are more active if they live closer to public transport suggesting they may be travelling to provision. This is in line with previous research [11], which has acknowledged how important transport infrastructure is, particularly when overcoming accessibility barriers [5, 9, 10].

This study provides evidence that improving activity and improving fitness are not intrinsically linked. Physical inactivity increases as activity does, especially for boys. Thus, MVPA cannot predict sedentary behaviour/time spent inactive or that being more inactive/sedentary cannot be a single determinant of poor activity, health and fitness [35]. Activities that are likely to influence activity, such as structured, competitive sports may have high periods of inactivity outside this formal training period. These could be the types of activities that boys are more likely to participate in outside of school. Research has shown that girls see high-intensity, competition as a barrier to being active [8]. This finding would suggest that we should promote different types of activity (e.g. light, moderate and vigorous) which have shown benefits to cardiovascular health and fitness, rather than restricting to focus on addressing sedentary behaviour [35]. Environments should improve access to and uptake of a variety of activities that promote beneficial physical activity, rather than simply aiming to reduce time spent inactive.

Interestingly, this study shows that more deprived teenagers are more fit. Moreover, additional analysis shows that girls are more active if they are from more deprived homes. It might be that pupils in deprived schools spend more time doing active travel compared to those who are less deprived. Despite being less likely to engage in activity in the form of structured, competitive sports clubs, it may be that teens from more deprived areas engage more in active travel due to the cost of running a car or getting public transport [36]. Girls, in particular, may enjoy active travel over other types of activity. Motivation was a significant factor in increased fitness in girls, therefore enjoyment is a key principal to focus on when prompting fitness and activity to this group. With this in mind, schools should focus on promoting and maintaining active travel and active travel infrastructure. As shown by previous research, the importance of promoting different types activity in young people cannot be overstated [35, 37, 38].

Generally speaking, over half of the teenagers involved in this study met the recommendation of 60 minutes of moderate-to-vigorous activity across the week which is high compared to previous data [2]. This could be due to the smaller sample size and consent rates to participate being higher in those more interested in being active. The prominence of the school setting in all outcomes highlights that to improve MVPA and fitness, interventions should centre on the school as a hub for teenagers as this study has shown it as a crucial setting. Where most environment-based studies and interventions focus on the home [4, 9, 13, 39–41], these findings suggest that focus is due on environments around the school to improve fitness and teenagers motivation to be active.

### Limitations

This study was only able to measure and ask the opinions of teenagers who consented, and this group may have been more interested in being active and more motivated to be active. Therefore, this study may investigate outcomes of predominantly more active teenagers. Furthermore, this study reports the findings from south Wales, which may not be generalizable to the whole population. Future work should include a larger sample size.

Future study should explore how teenagers travel around their environments; be it by foot, bicycle, car or public transport. This would help provide further context to the time spent in sedentary/MVPA. For example, some teenagers may be more inactive if they are travelling by car to provision rather than travelling by foot or cycling. Moreover, the study’s inclusion criteria for accelerometry analysis required participants to wear the device on 3 or more days regardless of weekday or weekend. Future studies should look to include at least one weekend day to note any difference between activity levels throughout the week.

GIS accessibility measures were developed using Euclidean distances which provides an indicative measure of access. In addition, access to public transport is based on bus stop locations as opposed to more sophisticated origin-destination measures. Further work could include more sophisticated network measures of access which take into account urban morphology and whether a destination (e.g. leisure centre) is served by a public transport route from an origin (e.g. home or school).

## Conclusions

This study highlights the importance of the school setting in improving MVPA and particularly, fitness for teenagers. Access to public transport, active travel infrastructure, access to green space and bringing activities that teenagers want and need closer to schools could make a significant difference to teenage health. Therefore, policy-makers/planning committees need to consider these provisions when designing teenage friendly environments namely, creating opportunities for teenagers to walk/cycle independently to activity provision around their homes and schools. Additionally, school communities could be utilised to support teenagers to make better use of these spaces when formal learning ends.

Environments that improve PA and fitness for teenagers should focus on bringing activity provision that teenager’s want and need into local communities. This provision should include different types of activity (e.g. light, moderate and vigorous) rather than addressing sedentary behaviour as there is evidence there is conflict between improving MVPA and increasing periods of inactivity/sedentary time.

## Supporting information

S1 Table Correlation data between independent variables. (DOCX)

S2 Table Linear regression results for MVPA by boys. (DOCX)

S3 Table Linear regression results for MVPA by girls. (DOCX)

S4 Table Linear regression results for fitness by boys. (DOCX)

S5 Table Linear regression results for fitness by girls. (DOCX)
