# Supplementary material for: How does the built environment affect teenagers (aged 13–14) physical activity and fitness? A cross-sectional analysis of the ACTIVE Project
Source: PLoS One. 2020 Aug 19;15(8):e0237784. doi: 10.1371/journal.pone.0237784 (PMC7437860; doi:10.1371/journal.pone.0237784)
Supplement: S1 Table — (DOCX) [file pone.0237784.s001.docx]

**S1 Table. Correlation data between independent variables.**

|  | Home Deprivation | Home to Active Travel | Home to Public Transport | Home to Main Road | Home to Natural Resource | Home to Nearest Activity | Home to School | School Deprivation | School to Active Travel | School to Public Transport | School to Main Road | School to Natural Resource | School to Nearest Activity |
| --- | --- | --- | --- | --- | --- | --- | --- | --- | --- | --- | --- | --- | --- |
| Home Deprivation | 1 |  |  |  |  |  |  |  |  |  |  |  |  |
| Home to Active Travel | 0.03 | 1 |  |  |  |  |  |  |  |  |  |  |  |
| Home to Public Transport | 0.01 | -0.01 | 1 |  |  |  |  |  |  |  |  |  |  |
| Home to Main Road | 0.16 | -0.08 | 0.21 | 1 |  |  |  |  |  |  |  |  |  |
| Home to Natural Resource | 0.08 | -0.11 | 0.07 | 0.19 | 1 |  |  |  |  |  |  |  |  |
| Home to Nearest Activity | -0.05 | -0.07 | 0.01 | 0.04 | 0.07 | 1 |  |  |  |  |  |  |  |
| Home to School | 0.03 | -0.24 | 0.31 | 0.06 | 0.05 | 0.1 | 1 |  |  |  |  |  |  |
| School Deprivation | 0.29 | 0.09 | -0.05 | 0.04 | 0.17 | 0 | -0.19 | 1 |  |  |  |  |  |
| School to Active Travel | 0.2 | -0.02 | -0.07 | 0.16 | 0.02 | -0.06 | 0 | 0.68 | 1 |  |  |  |  |
| School to Public Transport | -0.11 | -0.19 | -0.05 | 0.07 | -0.25 | -0.08 | 0 | -0.02 | 0.38 | 1 |  |  |  |
| School to Main Road | 0.22 | -0.08 | -0.05 | 0.21 | 0.22 | 0.01 | 0.26 | 0.09 | 0.46 | -0.21 | 1 |  |  |
| School to Natural Resource | -0.11 | -0.19 | 0.02 | -0.06 | 0.48 | 0.06 | 0.173 | -0.19 | -0.3 | -0.56 | 0.34 | 1 |  |
| School to Nearest Activity | 0.09 | -0.13 | -0.05 | -0.01 | 0.43 | 0.07 | -0.06 | 0.68 | 0.19 | -0.12 | -0.03 | 0.34 | 1 |
