# Supplementary material for: How does the built environment affect teenagers (aged 13–14) physical activity and fitness? A cross-sectional analysis of the ACTIVE Project
Source: PLoS One. 2020 Aug 19;15(8):e0237784. doi: 10.1371/journal.pone.0237784 (PMC7437860; doi:10.1371/journal.pone.0237784)
Supplement: S2 Table — (DOCX) [file pone.0237784.s002.docx]

**S2 Table. Linear regression results for MVPA by boys.**

| MVPA | Coef. | 95% Confidence Interval | p-value |
| --- | --- | --- | --- |
| Home Deprivation | 0.003 | -0.002 to 0.009 | 0.278 |
| Home to Active Travel | -0.001 | -0.003 to 0.003 | 0.743 |
| Home to Public Transport | 0.017 | -0.021 to 0.055 | 0.394 |
| Home to Main Road | 0.003 | -0.003 to 0.009 | 0.437 |
| Home to Natural Resource | -0.002 | -0.006 to 0.002 | 0.332 |
| Home Nearest Activity | 0.000 | -0.002 to 0.002 | 0.884 |
| Home to School | -0.001 | -0.001 to 0.001 | 0.308 |
| School Deprivation | 0.025 | -0.013 to 0.063 | 0.196 |
| School To Active Travel | -0.016 | -0.046 to 0.016 | 0.327 |
| School To Public Transport | 0.272 | 0.067 to 0.478 | 0.010* |
| School To Main Road | 0.003 | -0.017 to 0.023 | 0.768 |
| School To Natural Resource | 0.021 | 0.003 to 0.040 | 0.022* |
| School Nearest Activity | -0.012 | -0.029 to 0.006 | 0.177 |
| Distance Run | 0.001 | -0.006 to 0.008 | 0.851 |
| Sedentary Time | 0.071 | 0.039 to 0.103 | 0.000* |
| Motivation | -0.151 | -0.814 to 0.512 | 0.653 |

*Indicates significance.
