# Supplementary material for: How does the built environment affect teenagers (aged 13–14) physical activity and fitness? A cross-sectional analysis of the ACTIVE Project
Source: PLoS One. 2020 Aug 19;15(8):e0237784. doi: 10.1371/journal.pone.0237784 (PMC7437860; doi:10.1371/journal.pone.0237784)
Supplement: S3 Table — (DOCX) [file pone.0237784.s003.docx]

| MVPA | Coef. | 95% Confidence Interval | p-value |
| --- | --- | --- | --- |
| Home Deprivation | -0.012 | -0.019 to -0.003 | 0.008* |
| Home to Active Travel | -0.001 | -0.005 to 0.002 | 0.585 |
| Home to Public Transport | -0.007 | -0.013 to -.000 | 0.049* |
| Home to Main Road | -0.003 | -0.014 to 0.009 | 0.636 |
| Home to Natural Resource | 0.001 | -0.007 to 0.009 | 0.839 |
| Home Nearest Activity | -0.001 | -0.003 to 0.001 | 0.388 |
| Home to School | 0.003 | -0.001 to 0.006 | 0.175 |
| School Deprivation | 0.035 | -0.016 to 0.086 | 0.184 |
| School To Active Travel | -0.030 | -0.064 to 0.004 | 0.082 |
| School To Public Transport | 0.197 | -0.042 to 0.436 | 0.105 |
| School To Main Road | 0.022 | -0.001 to 0.046 | 0.071 |
| School To Natural Resource | 0.005 | -0.024 to 0.034 | 0.749 |
| School Nearest Activity | -0.013 | -0.039 to 0.013 | 0.333 |
| Fitness | -0.007 | -0.022 to 0.008 | 0.391 |
| Sedentary Time | 0.021 | -0.028 to 0.069 | 0.406 |
| Motivation | 0.017 | -0.749 to 0.784 | 0.965 |

**S3 Table. Linear regression results for MVPA by girls.**

*Indicates significance.
