# Supplementary material for: How does the built environment affect teenagers (aged 13–14) physical activity and fitness? A cross-sectional analysis of the ACTIVE Project
Source: PLoS One. 2020 Aug 19;15(8):e0237784. doi: 10.1371/journal.pone.0237784 (PMC7437860; doi:10.1371/journal.pone.0237784)
Supplement: S4 Table — (DOCX) [file pone.0237784.s004.docx]

| Distance Run | Coef. | 95% Confidence Interval | p-value |
| --- | --- | --- | --- |
| Home Deprivation | 0.049 | -0.083 to 0.181 | 0.466 |
| Home to Active Travel | 0.027 | -0.050 to 0.104 | 0.491 |
| Home to Public Transport | 0.770 | -0.146 to 1.686 | 0.099 |
| Home to Main Road | -0.001 | -0.153 to 0.151 | 0.993 |
| Home to Natural Resource | -0.034 | -0.132 to 0.064 | 0.5 |
| Home Nearest Activity | -0.006 | -0.062 to 0.051 | 0.839 |
| Home to School | -0.002 | -0.031 to 0.027 | 0.878 |
| School Deprivation | -0.568 | -1.482 to 0.345 | 0.221 |
| School To Active Travel | 0.305 | -0.439 to 1.049 | 0.419 |
| School To Public Transport | -3.258 | -8.244 to 1.728 | 0.198 |
| School To Main Road | 0.115 | -0.365 to 0.595 | 0.636 |
| School To Natural Resource | -0.344 | -0.787 to 0.099 | 0.127 |
| School Nearest Activity | 0.333 | -0.085 to 0.751 | 0.117 |
| MVPA | 0.386 | -3.670 to 4.443 | 0.851 |
| Sedentary Time | -0.473 | -1.272 to 0.326 | 0.244 |
| Motivation | 15.444 | -0.158 to 31.045 | 0.052 |

**S4 Table. Linear regression results for Fitness by boys.**

*Indicates significance.
