# Supplementary material for: How does the built environment affect teenagers (aged 13–14) physical activity and fitness? A cross-sectional analysis of the ACTIVE Project
Source: PLoS One. 2020 Aug 19;15(8):e0237784. doi: 10.1371/journal.pone.0237784 (PMC7437860; doi:10.1371/journal.pone.0237784)
Supplement: S5 Table — (DOCX) [file pone.0237784.s005.docx]

| Distance Run | Coef. | 95% Confidence Interval | p-value |
| --- | --- | --- | --- |
| Home Deprivation | -0.059 | -0.186 to 0.069 | 0.362 |
| Home to Active Travel | 0.020 | -0.038 to 0.078 | 0.501 |
| Home to Public Transport | -0.006 | -0.107 to 0.095 | 0.908 |
| Home to Main Road | -0.120 | -0.293 to 0.053 | 0.172 |
| Home to Natural Resource | 0.020 | -0.100 to 0.140 | 0.744 |
| Home Nearest Activity | 0.012 | -0.026 to 0.050 | 0.536 |
| Home to School | 0.024 | -0.030 to 0.078 | 0.383 |
| School Deprivation | -0.047 | -0.809 to 0.715 | 0.902 |
| School To Active Travel | -0.124 | -0.636 to 0.387 | 0.63 |
| School To Public Transport | 1.059 | -2.484 to 4.601 | 0.554 |
| School To Main Road | 0.416 | 0.0655 to 0.766 | 0.021* |
| School To Natural Resource | -0.081 | -0.514 to 0.351 | 0.709 |
| School Nearest Activity | 0.080 | -0.310 to 0.471 | 0.684 |
| MVPA | -1.418 | -4.689 to 1.853 | 0.391 |
| Sedentary Time | -0.264 | -0.980 to 0.453 | 0.466 |
| Motivation | 16.777 | 6.251 to 27.302 | 0.002* |

**S5 Table. Linear regression results for Fitness by girls.**

*Indicates significance.
